# Brugada Phenocopy Induced by Recreational Drug Use

**DOI:** 10.1155/2018/6789253

**Published:** 2018-04-11

**Authors:** Adedoyin Akinlonu, Ranjit Suri, Priyanka Yerragorla, Persio D. López, Tuoyo O. Mene-Afejuku, Olatunde Ola, Carissa Dumancas, Jumana Chalabi, Gerald Pekler, Ferdinand Visco, Savi Mushiyev

**Affiliations:** ^1^Department of Medicine, Health+Hospitals/Metropolitan, New York Medical College, Valhalla, NY, USA; ^2^Cardiovascular Department, Arrhythmia Services Mount Sinai Health System Hospitals, Mount Sinai St. Luke's, New York, NY, USA; ^3^Department of Medicine, Division of Cardiology, Health+Hospitals/Metropolitan, New York Medical College, Valhalla, NY, USA

## Abstract

Recreational drugs are commonly abused in all age groups. Intoxication with these substances can induce silent but significant electrocardiographic signs which may lead to sudden death. In this case study, we present a 49-year-old male with no medical comorbidities who came to the emergency department requesting opioid detoxification. Toxicology screen was positive for cocaine, heroin, and cannabis. Initial electrocardiogram (EKG) showed features of a Brugada pattern in the right precordial leads, which resolved within one day into admission. This presentation is consistent with the recently recognized clinical entity known as Brugada phenocopy.

## 1. Introduction

The Brugada electrocardiographic pattern (BEP) is a coved-type, downsloping, ≥2 mm ST segment elevation with T-wave inversion among the right precordial leads V1-V2 (occasionally only in V1 and unusually also in V3) termed type 1 BEP, or a saddle-back convex ST segment elevation with variable T wave in V1, and positive or flat T wave in lead V2 (types 2 or 3 BEP) [1]. Initial guidelines required documented, polymorphic ventricular tachycardia or fibrillation, a family history of sudden cardiac death (≤45 years old), coved-type electrocardiograms in family members, syncope, or nocturnal agonal respiration in addition to the type 1 BEP for a diagnosis of Brugada syndrome (BrS) [2–4]. The latest guidelines, nonetheless, only require the documentation of type 1 BEP, either spontaneously or as a consequence of a provocative test with sodium channel blockers for a diagnosis of BrS [5].

## 2. Case Presentation

A 49-year-old Hispanic man presented to our institution requesting opioid detoxification. He had no specific clinical complaints, and the review of systems was negative. The patient's physical examination was unremarkable. Initial workup revealed normal complete blood count, electrolyte panel, and renal and hepatic function; a urine toxicology screen was positive for cocaine, opioid, and cannabinoid use.

**Figure 1:**
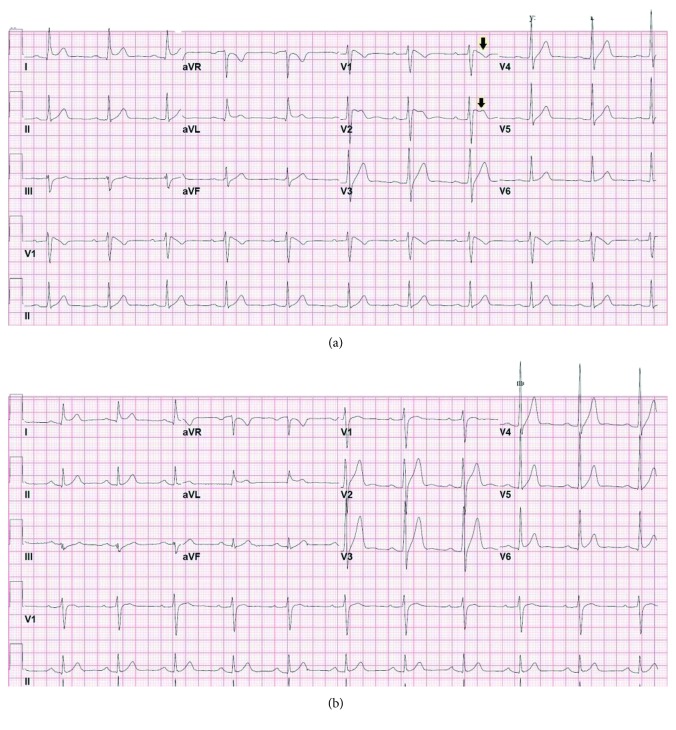
Electrocardiograms of the first admission revealing transient Brugada pattern (a, arrow) with complete resolution the following morning (b).

**Figure 2:**
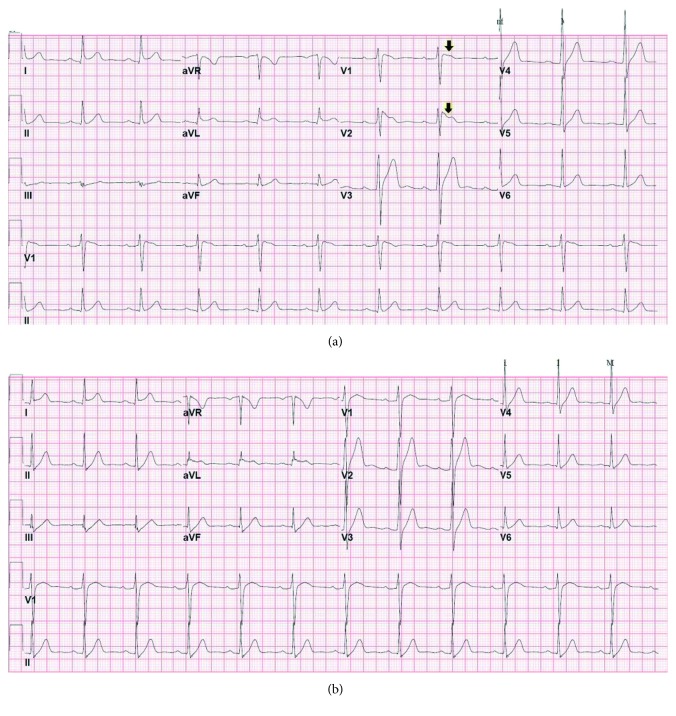
Electrocardiograms revealing recurrence of the Brugada pattern in the setting of recreational drug use (a, arrow) and complete resolution after approximately 24 hours without substance use (b).

An electrocardiogram obtained on admission (Figure 1(a)) revealed a Brugada pattern which was not present on previous studies. He was monitored on a telemetry unit, and a repeat electrocardiogram the morning after showed complete resolution of the Brugada-type abnormalities (Figure 1(b)). On targeted interrogation, he denied any syncopal episodes and he had not been informed of nocturnal breathing abnormalities by his partner; there had been no sudden, unexplained deaths in his family. The patient's parents were contacted, and their electrocardiograms were found to be normal. Since the patient's electrocardiogram was not completely normal, he was referred to an affiliated tertiary institution for provocative testing with sodium channel blockers; however, he did not follow-up. Multiple attempts to convince the patient to undergo further workup were unsuccessful. Five months later, he was readmitted to the observation unit of our institution for evaluation of cocaine-associated chest pain. The urine toxicology screen was again positive for cocaine, opioid, and cannabinoid use. His admission electrocardiogram revealed recurrence of the Brugada pattern (Figure 2(a)), which resolved after 24 hours (Figure 2(b)). Both a transthoracic echocardiogram and a stress myocardial perfusion scintigraphy were normal. Once more, he declined further workup. The patient was asymptomatic and hemodynamically stable at the time of hospital discharge.

## 3. Discussion

BrS is an autosomal dominant, low-penetrance disease caused by several different loss-of-function gene mutations that affect the Na_v_1.5 sodium channel [6]. In addition, mutations causing loss of function of the calcium channel currents and a gain of function of potassium channel currents have also been associated with BrS [7]. These genetic abnormalities are thought to induce defective depolarization and/or early repolarization [8, 9], which lead to the clinical manifestations of BrS: life-threatening arrhythmias and sudden cardiac death.

Not all patients with BrS, however, become symptomatic [6], which has led to recommendations regarding risk stratification [7]. Moreover, BEP has been reported in association to several acute and persistent conditions: acute coronary events, pericarditis, myocarditis, pulmonary embolism, metabolic disorders, electrolyte imbalances, dissecting aorta aneurysm, thiamine deficiency, electric shock, certain pharmacologic agents, left ventricular hypertrophy, athlete's heart, right bundle branch block, pectus excavatum, septal hypertrophy, arrhythmogenic right ventricular cardiomyopathy or dysplasia, autonomic nervous system abnormalities, Duchenne dystrophy, Friedreich's ataxia, mediastinal tumor, and Chagas disease [7, 10].

Recently, the term “Brugada phenocopy” (BrP) has been used to describe these cases, provided they fulfill the following criteria: there is an identifiable trigger, BEP resolves after removal of said trigger (when possible), and the results of a provocative test with sodium channel blockers are negative [10]. Patients with BrP would be classified morphologically according to the type of BEP and further qualified depending on whether all diagnostic criteria are met [10, 11]. The term is still controversial, as it has been argued that data regarding a genetic predisposition are lacking in almost all cases [7]. We believe it is a helpful descriptor to identify patients with a lower risk of developing the feared complications of BrS that would warrant invasive therapeutics.

Both cannabis and cocaine have been associated with BrP [12–14], possibly due to their activities on the cardiac voltage-gated sodium channel [14, 15]. The EKG changes seen with cannabis use are a result of its action as a partial agonist of the cardiac sodium channels, and arrhythmias can be induced if the action potential is shortened [12, 13, 15]. Cocaine, on the other hand, blocks sodium and potassium ion channels and also inhibits calcium entry into the cardiac myocytes thereby impairing action potential and altering the normal electrical activity of the heart [9, 16]. A synergistic effect is observed by coingestion of the above two drugs since cannabis increases the plasma concentration of cocaine [16]. Heroin has also been associated with BEP, though the exact cardiovascular mechanism remains to be described [16, 17]. A combination of the effects of these substances likely triggered the appearance of BEP in our patient. Unlike in symptomatic patients, individuals with drug-induced Brugada EKG pattern who are asymptomatic generally have good prognosis (i.e., there is a low risk of lethal arrhythmias), and the observed EKG changes is likely to be benign [9, 18]. Therefore, the need for provocative testing in these groups of patients might be unwarranted but reasonable to pursue when feasible with due considerations given to the risks and benefits [11, 18].

This presentation of highly suspected BrP in the setting of cocaine, cannabinoid, and heroin use continues to raise the alarm about the cardiovascular effects of these substances. Although the clinical implications of BrP remain to be elucidated, genetic testing may help in differentiating BrP from BrS and thus with managing these patients.
